# Soil phosphorus availability as affected by root exudates of cover crop species

**DOI:** 10.1038/s41598-025-19102-7

**Published:** 2025-09-29

**Authors:** Tamjid Us Sakib, Nathan O. Nelson, Ganga M. Hettiarachchi, Colby J. Moorberg, Jesse B. Nippert, Susan Whitaker

**Affiliations:** 1 Department of Agronomy, Kansas State University, 2004 Throckmorton Plant Sciences Center 1712 Claflin Rd., Manhattan, KS 66506 USA; 2 Division of Biology, Kansas State University, 209 Bushnell Hall 1717 Claflin Rd, Manhattan, KS 66506 USA; 3 Department of Biochemistry and Molecular Biophysics, Kansas State University, 141 Chalmers Hall 1711 Claflin Rd, Manhattan, KS 66506 USA

**Keywords:** Root exudates, Low molecular weight organic acids, P availability, Cover crop species, And plant family, Environmental chemistry, Environmental chemistry, Environmental sciences, Ecology, Agroecology

## Abstract

Plant species can modify the phosphorus (P) sorption in soils through the release of low molecular weight organic acids (LMWOA) in their root exudates. These LMWOAs compete with P for adsorption sites on soil minerals and induce dissolution of Fe and Al oxyhydroxides via complexation or ligand promoted dissolution, which could affect P availability for crops and P loss. This study examined the effect of plant species and P input on the P sorption dynamics with respect to LMWOA. A greenhouse study was conducted with ten different plant species, two P fertilizer treatments (0 and 70 kg P ha^− 1^), and two-time intervals (35 and 70 days). All treatments were structured in a 10 × 2 × 2 complete factorial with two controls arranged in a randomized complete block design with five replicates (210 experimental units). Species from *Poaceae* and *Fabaceae* exhibited a consistent trend of increased LMWOA release in low P compared to high P conditions whereas *Brassicaceae* species consistently released higher concentrations of LMWOAs regardless of P. On average, LMWOA release followed the order: *Brassicaceae > Fabaceae > Poaceae*. In certain circumstances, P availability may be more closely related to specific LMWOAs, particularly oxalic and citric acids, rather than total LMWOA concentration. Their influence on P sorption and water-extractable P is dependent on plant species, growth stage and P status. Our findings suggest that certain cover crop species rye, triticale and crimson clover may have potential to enhance P availability as well as minimize P loss.

**Supplementary Information:**

The online version contains supplementary material available at 10.1038/s41598-025-19102-7.

## Introduction

Low molecular weight organic acids (LMWOAs) in root exudates have potential to increase P mobility in soils via competitive adsorption and ligand promoted dissolution^1,2^. Many studies investigating LMWOAs have been conducted using nutrient solutions, sterilized sand, or soils, often involving a limited number of plant species. Among the studies conducted with soils, most have assessed the effects of LMWOAs on P availability by measuring plant growth parameters and P uptake^3–7^. However, these approaches often do not establish a direct link between LMWOA release from plant roots and soil P availability through sorption and desorption processes. Even in recent soil-based studies, the emphasis tends to be on P speciation rather than mechanistic insights into soil P sorption dynamics^8–10^. Studies based on simplified systems or soil analyses without investigating P sorption mechanisms may offer only a partial view of the role LMWOAs play in modifying P availability. Consequently, our understanding of how LMWOA release from plant roots directly influences soil P sorption and desorption in soil remains limited. Additionally, the methodologies for collection and analysis of LMWOAs varied across studies^11^. Therefore, it is necessary to obtain more information on root exudates of different crop species and their effect on P dynamics in natural soils.

Oxalic, malic, citric, succinic, tartaric, fumaric are simple organic acids exuded by plant roots and are commonly found in soils^12,13^. The concentrations of total LMWOAs in the rhizosphere or in soil solutions vary greatly, ranging from 10 to 20 µM to over 80 mM^14,15^. Although these LMWOAs are readily absorbed by solid phases or are taken up by microorganisms, their continual production makes them chemically important. Continuous addition of LMWOAs, even at low concentrations, can enhance soil P availability by promoting desorption of native P through complexation or ligand-promoted dissolution Fe and Al oxyhydroxides. Additionally, LMWOAs can increase availability of added P through competitive adsorption, where LMWOAs occupy adsorption sites and thereby reduce P adsorption. These processes highlight the important role of LMWOAs in regulating soil P dynamics. When LMWOAs and soluble P concentrations in soil solution exceed the soil adsorption capacity, or LMWOAs enhance dissolution of Fe and Al oxyhydroxides in soil, the excess or released P becomes available for plant uptake and could increase the potential for P loss in runoff water.

The ability of organic anions to affect P solubility and reduce P sorption depends on their molecular structure and soil pH. Tricarboxylic acids are generally more effective than dicarboxylic acids, while monocarboxylic acids have minimal effect on P solubility^15^. Several studies have reported that citrate and oxalate can form stable complexes with the Fe, Al and Ca in soils, thereby reducing P sorption. Both organic anions can be specifically absorbed on both Al and Fe-oxide surfaces of soils and therefore replace P bound to these surfaces through a ligand-exchange reaction or even dissolve calcium phosphates to release Earl et al.^16^ and Cajuste et al.^17^ found that 10 mM of citric acid in systems can reduce P sorption by almost 50%. The reduction of P sorption indicates that a certain amount of P adsorbing sites in the soil were eliminated by organic acids. Fox and Kamprath^18^ found that organic acids could increase the P concentration in soil solution by 10–1000 times. Furthermore, Biswas et al.^19^ reported that oxalic acid can mobilize 37% more plant-available P compared to soils without oxalic acid addition. Similarly, Khademi et al.^20^ demonstrated that organic acids play a significant role in mobilizing soil P. Their study showed that concentrations of 1 mM oxalic and citric acid released approximately 1–2.5 mg P kg^− 1^ soil, while 10 mM concentrations released 10–25 mg P kg^− 1^ soil. These findings collectively suggest that root-released organic anions, such as oxalic and citric acid, have strong potential to enhance soil P availability. These organic anions in root exudates act as natural chelators, enhancing P solubility through ligand-promoted dissolution by forming stable, soluble metal-organic acid complexes via inner-sphere complexation. Among organic anions, citrate exhibits a higher affinity for Fe³⁺ and Al³⁺ than oxalate due to its greater stability constants, facilitating the formation of more stable complexes that can enhance P availability^21,22^.

Diverse crop species are used as cover crops in agricultural systems to enhance soil health, reduce sediment and nutrient loss, and stimulate nutrient cycling^23,24^. Studies have revealed that using cover crops effectively reduces erosion, but may increase dissolved reactive P loss in runoff water^25^. This might be due to cover crop effects on P sorption through influencing processes like competitive adsorption and ligand promoted dissolution in soil systems. At present, the underlying processes responsible for cover crops species influence on P availability is unknown. The selection of species as cover crops depends on climates, cropping systems and their biomass production^26^. The composition of root exudates varies by species due to inherent physiological processes^27^. Therefore, the rhizosphere properties of different plant species can differ based on root exudate composition. It is possible that plant species belonging to specific plant families can enhance their P availability by releasing LMWOA. Species from *Fabaceae* and *Brassicaceae* can exude P mobilizing compound that is LMWOA, which make the recalcitrant P available for the subsequent crop^28–30^. Furthermore, plant species from *Poaceae*, such as rye (*Secale cereale*) and wheat (*Triticum aestivum*), can also use less labile P fractions through different acquisition strategies and accumulate it in their aboveground plant parts^31^. This indicates that P availability varies across plant species due to their distinct physiological processes involving organic anions, which can impact P availability. The soluble P in soil solution through P acquisition strategies is available for both plant uptake as well as for potential P loss^32,33^. Therefore, information on LMWOA release is important when choosing species for cover crops for soil health or water quality protection. However, there is not enough information available for commonly used cover crop species in terms of LMWOA release.

Previously, most studies were conducted using synthetic LMOWAs or clay complexes, that didn’t provide enough information about naturally produced and released LMWOA effects on P sorption in field soils. Additionally, most studies focused on just identifying and quantifying LMWOAs without linking their release to soil P sorption properties, thus compromising our understanding of LMWOA effects on P dynamics. Therefore, it is crucial to study the amount and types of LMWOAs released from different crop species belonging to various plant families and their effects on P sorption dynamics in the soil. This research will improve our understanding of the mechanisms involved in LMWOA effects on the adsorption-desorption and precipitation-dissolution processes of soil P.

We hypothesized that (1) plant species and family with greater LMWOA release in root exudates will increase P availability in soils through ligand-promoted dissolution of mineral bound P, thereby increasing water extractable P (WEP) in soils, and (2) plant species and family with greater LMWOA release in root exudates will decrease P sorption in soil through competitive sorption processes, thereby enhancing the availability of P released from soil or fertilizer sources. In that context, our objectives are to (1) identify and quantify the LMWOAs in root exudates of different crop species and (2) determine the effect of root exudates on P availability to understand the soil processes influencing P sorption with respect to P input and cover crop growth.

## Materials and methods

### Soil collection

Soil was obtained from 0 to 10 cm of the Ap horizon (weak fine granular structure) of a Smolan silty clay loam (fine, smectitic, mesic Pachic Argiustoll) according to USDA soil taxonomy. All surface residue was removed prior to collection, following which the soil was air-dried, passed through a 4-mm Zn-free stainless-steel mesh/sieve, and stored in five portions for greenhouse experiments (one for each replication). A sub-sample of soil was removed for each replicate, ground to pass a 2-mm sieve, and analyzed for the following: soil texture, pH, total organic carbon (TOC), soil test P (STP), total nitrogen, exchangeable cations (K, Ca, Mg, Na), and cation exchange capacity (CEC) (Table 1). All initial analyses were conducted by the Soil Testing Laboratory at Kansas State University.

**Table 1:** Soil properties of silty clay loam soil used in greenhouse study.

Parameters	Value (± SE)
pH	6.9 (± 0.07)
Sikora pH	7.3 (± 0)
Mehlich 3 P, mg kg^−1^	4.6 (± 1.95)
Total N, g kg^−1^	1.6 (± 0.14)
NO_3_-N, mg kg^−1^	7.06 (± 2.84)
NH_4_-N, mg kg^−11^	5.96 (± 1.43)
Total organic carbon (TOC), g kg^−1^	10.62 (± 1.7)
Exchangable K, mg kg^−1^	177 (± 23.73)
Cation exchange capacity (CEC) cmol kg^−1^	18.24 (± 0)
Exchangeable Ca, mg kg^− 1^	3093 (± 169.07)
Exchangeable Mg, mg kg^−1^	355 (± 14.95)
Exchangeable Na, mg kg^−1^	10 (± 1.48)
Soil texture	Silty clay loam

Soil texture was determined using the hydrometer method, pH was measured in a 1:10 soil: water suspension, and buffer pH was determined using the Sikora method^34^. Soil test P (STP) was assessed using the Mehlich−3 method^35^, TOC content was measured through a dry combustion method^36^ and soil inorganic nitrogen (NH_4_−N and NO_3_−N) was determined after extraction with KCL^37^. Exchangeable cations extracted with ammonium acetate were measured using ICP−OES, and cation exchange capacity was determined through the summation method. Phosphorus in the Mehlich 3 extract was determined with the molybdate−blue colorimetric method.

### Experimental design

Ten different crop species were selected for this study, comprising species from the *Poaceae*, *Fabaceae* and *Brassicaceae* families that are commonly used for both grain production (field crops) and improving soil health (cover crops) in the USA Midwest region (Table 2). Two P fertilizer application rates of 0 and 70 kg P ha^− 1^, which can be denoted by No-P and P addition respectively throughout the paper were selected to evaluate the effect of released LMWOAs on soil P sorption processes. The No-P rate was chosen to simulate P-deficient conditions where LMWOAs are expected to enhance P availability primarily through ligand-promoted dissolution and increased desorption of native soil P whereas P addition rate represents high P conditions, where LMWOAs may increase P availability by competing with phosphate ions for sorption sites, thereby decreasing P adsorption. Two-time intervals, 35 days and 70 days, were chosen to investigate their effects. The crop species, P treatment rates, and time interval treatments were organized in a 10 × 2 × 2 complete factorial treatment structure arranged in a randomized complete block design and replicated five times. Additionally, each replication included two controls (without the inclusion of any plant species): one pot received 0 kg P ha^− 1^, while another pot received 70 kg P ha^− 1^. Consequently, a total of 210 experimental units were established.

**Table 2:** Plant species used in the study.

Family	Common name	Scientific name	Seedling rate	Final plant population after thinning (plants per pot)
*Poaceae*​	Corn​	*Zea Mays*​	64,000 count/ha	1
	Wheat​	*Triticum Aestivum*​	84 g/ha	12
	Triticale​	*Triticosecale*​	84 g/ha	12
	Cereal rye​	*Secale cereale*​	67 g/ha	12
*Fabaceae*​	Soybean​	*Glycine Max*​	325,000 count/ha	3
	Crimson clover​	*Trifolium incarnatum*​	17 g/ha	15
	Sunn hemp​	*Crotalaria juncea*​	11 g/ha	8
	White lupin​	*Lupinus albus*​	56 kg/ha	5
*Brassicaceae*​	Rapeseed​	*Brassica napus*​	6 g/ha	15
	Turnip​	*Brassica rapa*​	2 g/ha	15

### Greenhouse experiment

The research was conducted at the Kansas State University greenhouse facility in Manhattan, Kansas, USA. For the P addition treatment, 2-kg of soil was amended with 0.55 g of crushed Triple Super Phosphate (N-P_2_O_5_-K_2_O; 0-46-0) fertilizer which was approximately 70 kg P ha^− 1^, mixed for 3 min in a V-shell mixer, and placed in a plastic pot (14 cm diameter by 17.78 cm height). For the No-P treatment, 2 kg of soil (dry weight-basis) were placed directly in the pots. In both P cases, soil was added pots at a bulk density of 1.08 g cm^− 3^. After filling, each pot received 500 ml of tap water through drip irrigation at a rate of 1.89 L/h as an initial wetting. Subsequently, the soil was allowed to drain and equilibrate for 24 h. Initial wetting of the soil was carried out to prevent water channeling within the pot. Following the wetting process, seeds were manually placed on the soils using the hand drilling method. The seed rate per pot for each crop species was determined based on the recommendations of the Midwest Cover Crops Council^38^. Nitrogen was uniformly applied to all pots at a rate of 200 mg N kg^− 1^ soil in the form of NH_4_NO_3_. Half of the nitrogen was added with 100 ml of water on the day of seeding (to bring soil initial moisture to 0.30 cm^3^ cm^− 3^), while the remaining dose was applied after 4 weeks of growth along with irrigation.

After emergence, seedlings were thinned as per the guidelines for seed rate by Midwest Cover Crops Council. Pot moisture was monitored visually, and all plants were irrigated approximately every two days with 200 ml of tap water through drip irrigation. Throughout the experiment, plants were exposed to natural daylight (13 h) and maintained at a constant air temperature of 21 °C during the day and 18 °C at night. Although the plant species studied have different optimal temperatures for growth, temperature was kept consistent to avoid introducing it as an additional treatment factor. This was important because temperature can influence soil chemical and biological processes, potentially affecting the role of naturally released LMWOAs in soil P sorption and desorption, and ultimately impacting P availability.

### Low molecular weight organic acid (LMWOA) extraction and analysis

Root exudates were extracted from plant roots at 35 and 70 days after planting based on the following protocol. Pots designated for the corresponding time interval were carefully removed from the greenhouse. By the end of the study, plant roots had extensively proliferated throughout the pots, forming a dense network that likely influenced the entire soil volume (Fig S1). The soil and root mass were removed from the pot and the soil adhering to the roots was removed by careful shaking. Subsequently, the root systems with adhering soil were immersed in a container with 200 ml of 0.2 mM CaCl_2_ solution and gently shaken for 30 s to obtain a rhizosphere extract^39–41^. A 20-mL aliquot of the extract was immediately removed and passed through a Millex 25MM prefilter and a 0.22 µM syringe filter in series. The rhizosphere extract was stored at − 20 °C until analysis^42^. Root samples were dried at 68 °C for about four days until they reached a constant mass. Plant roots used for the extraction were dried and weighed to present the total concentration of organic acids on a dry root weight basis. After separating the roots from the bulk soil in the pot, the soil was thoroughly mixed by hand and a subsample was air-dried, ground to pass a 2 mm-sieve, and saved for subsequent soil analysis (see below). Root separation from soil and soil collection were conducted when pot moisture was approximately 30%, and this level was consistently maintained across all treatments to minimize potential for differences in soil moisture to contribute to variability in these measurements.

A Quadrupole Time-of-Flight Mass Spectrometer (QToF MS) (Waters Xevo G2-XS QToF) was used to identify and quantify organic acids in the rhizosphere extracts. Eight distinct organic acids were chosen for identification and quantification based on common LMWOAs found in root exudates^43^ (Table 3). To prepare the standards, different concentrations (0.01, 0.1, 1.0, 10, 10, 100, 1000 µM) of organic acids were individually prepared (Table 3). Acetic acid with ^13^C isotope-labeled was introduced as the internal standard. A standard curve was prepared from the mixture of all eight different organic acids, and the concentration of each acid was calculated by comparing its intensity to the intensity of acetic acid. The limit of detection for each acid was determined according to methods of Long et al.^44^ and is presented in Table 3. Blank samples and internal standards were also included for Quality assurance/Quality control.

**Table 3:** Types of LMWOAs identified and their limit of detection (LOD) in mass spectrometer.

Organic acids identified		Standard chemicals used	LOD (µM)
Tricarboxylic acid	Citric	Thermo Scientific Citric acid monohydrate, 99.9%	0.0003
Dicarboxylic acid	Malic	Acros Organics Malic acid, 99%	0.0005
	Oxalic	Fisher chemical Oxalic acid dihydrate, 99.5%	0.44
	Tartaric	Fisher chemical Tartaric acid, 99%	0.0002
	Succinic	Spectrum Maleic acid,99-100.5%	0.000003
	Maleic	Spectrum Maleic acid,98–102%	0.002
	Fumaric	Acros Organics Fumaric acid, 99%	0.12
Monocarboxylic acid	Formic	Fisher chemical Formic acid, 99%	0.47

### Soil analysis

Water-extractable phosphorus (WEP) in soils was determined by extracting two grams of air-dried soil (≤ 2 mm) with 20 mL of distilled-deionized water in a 50 ml plastic centrifuge tube. Samples were shaken for one hour on an end-to-end shaker, then centrifuged at 10,000 rpm (15428 xg) for 10 min, and the supernatant was filtered through a 0.45 μm syringe filter^45^. The P concentration in the solution was determined using the molybdate-blue colorimetric method with a flow injection analyzer (Quick Chem 8500 Series II; Quick Chem Method 10-115-01-1-A, Lachat Instruments).

A single-point phosphorus (P) adsorption capacity was determined by combining two grams of air-dried soil (≤ 2 mm) with 20-ml of phosphate solution with 1.0 mg l^− 1^ P. Samples were placed in a 50-ml plastic centrifuge tube and shaken for 24 h on an end-to-end shaker. After centrifugation at 10,000 rpm (15428 xg) for 10 min, the solution was filtered through a 0.45 μm syringe filter. The equilibrium concentration in the solution was then analyzed using the molybdate-blue colorimetric method with a flow injection analyzer (Quick Chem 8500 Series II; Quick Chem Method 10-115-01-1-A, Lachat Instruments). The amount of P sorbed by the soil was subsequently calculated^46^.

Amorphous iron (Fe), aluminum (Al), and associated phosphorus (P) were determined through extraction with ammonium oxalate^47^. One gram of air-dried soil (≤ 2 mm) was mixed with 40 ml of 0.2 M Ammonium Oxalate in a 50 ml plastic centrifuge tube and shaken for 2 h on an end-to-end shaker in dark conditions. After centrifugation at 10,000 rpm (15428 xg) for 10 min, the solution was filtered through a 0.45 μm syringe filter. The concentration of Al, Fe, and P in the solution was then analyzed using Inductively Coupled Plasma Optical Emission Spectroscopy (ICP-OES). Soil pH was measured in a 1:1 soil: water suspension with double junction mercury electrode^48^.

### Data and statistical analysis

Total LMWOA concentration is the sum of concentration for all eight organic acids. These concentrations were expressed per gram of dry root mass. Potential outliers were detected with the modified Z-Scores^49^ and removed prior to analysis. The effects of P input, crop species, and their interaction on LMWOA concentration, WEP, and sorbed P were determined using ANOVA with SAS Proc Glimmix 9.4. To meet the assumptions of normally distributed residuals, some data (concentrations of all organic acids and WEP) were log-transformed. Back-transformed least squares mean is presented in results section for the transformed variables. Pairwise comparisons were conducted using protected LSD at a significant level of 0.05. Data on percent LMWOA distribution were transformed with the Arcsine transformation and means were back transformed for presentation. Moreover, Contrast statements were used in SAS to determine statistical differences between species grouped by taxonomic families.

## Results

### Total concentration of low molecular weight organic acid (LMWOA)

**Fig. 1:**
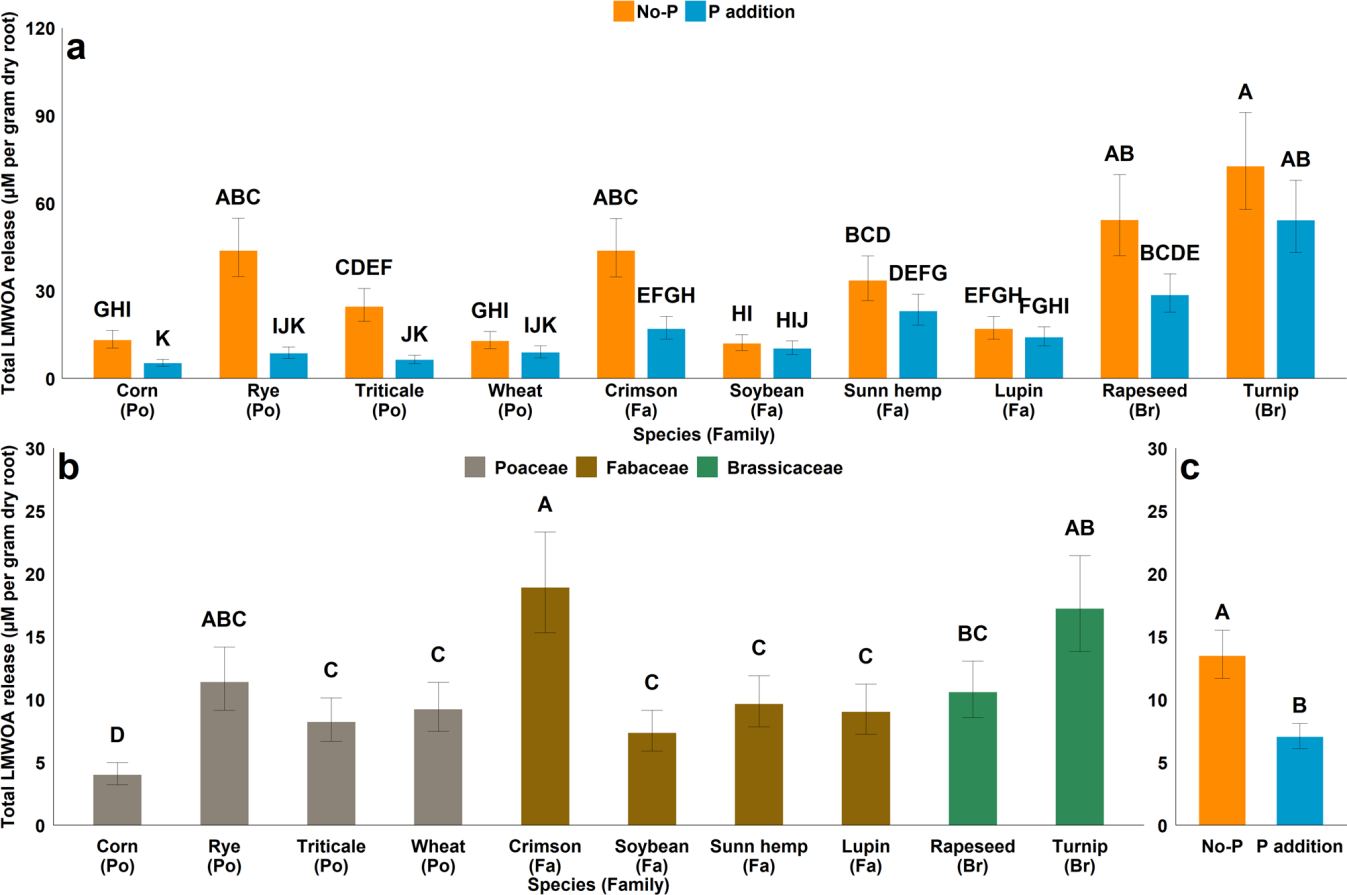
(**a**) effect of species × P interaction on total LMWOA release at 35 days after planting (**b**) main effect of species on total LMWOA release at 70 days after planting (**c**) main effect of P treatment on the total LMWOA release at 70 days after planting. (Po = *Poaceae*, Fa = *Fabaceae*, Br = *Brassicaceae*) Bar with different letters indicate significant differences between treatments.

At 35 days, the LMWOA release was affected by the main effects of both species and P, as well as their interaction (Table 4). Although the addition of P fertilizer tended to decrease total LMWOA release in all species, the decrease was significant in only a few species (corn, rye, triticale, and crimson clover) (Fig. 1a). The general order of LMWOA release based on plant families was observed as *Brassicaceae > Fabaceae > Poaceae*. The addition of P decreased LMWOA release for species belonging to *Poaceae* more than the other two families, as indicated by the contrast statements for the effect of P on LMWOA release (Table 4).

At 70 days, the LMWOA release was affected by both species and P, but not their interaction (Table 4). The LMWOA release was greater when plants were grown in soil without P addition than when soils received P addition (Fig. 1c). Corn released the least LMWOA, and crimson clover and Turnip were among the species with the greatest release (Fig. 1b), similar to trends observed at 35 days. The general order of LMWOA release after 70 days based on plant families was *Brassicaceae = Fabaceae > Poaceae*. The effect of P was the same for all plant families at 70 days (Table 4).

**Table 4:** ANOVA results assessing the effects of crop species and P treatment and their interaction for total organic acid release by plant roots and *p*-values from contrasts comparing species grouped by plant families.

Effect	*P* value	
Days	**35 days**	**70 days**
Species	**< 0.001**	**< 0.001**
P	**< 0.001**	**< 0.001**
Species × P	**0.008**	0.482
Contrasts comparing plant families		
Po vs. Fa	**< 0.001**	**0.017**
Po vs. Br	**< 0.001**	**< 0.001**
Fa vs. Br	**< 0.001**	0.111
Effect of P; Po vs. Br	**0.031**	0.425
Effect of P; Po vs. F	**0.003**	0.528
Effect of P; Fa vs. Br	0.847	0.778

P = phosphorus, Po = poaceae, Fa = Fabaceae Ba = brassicaceae.

Significant effects and interactions (*p* < 0.05) are indicated in bold.

### Distribution of low molecular weight organic acid (LMWOA)

**Fig. 2:**
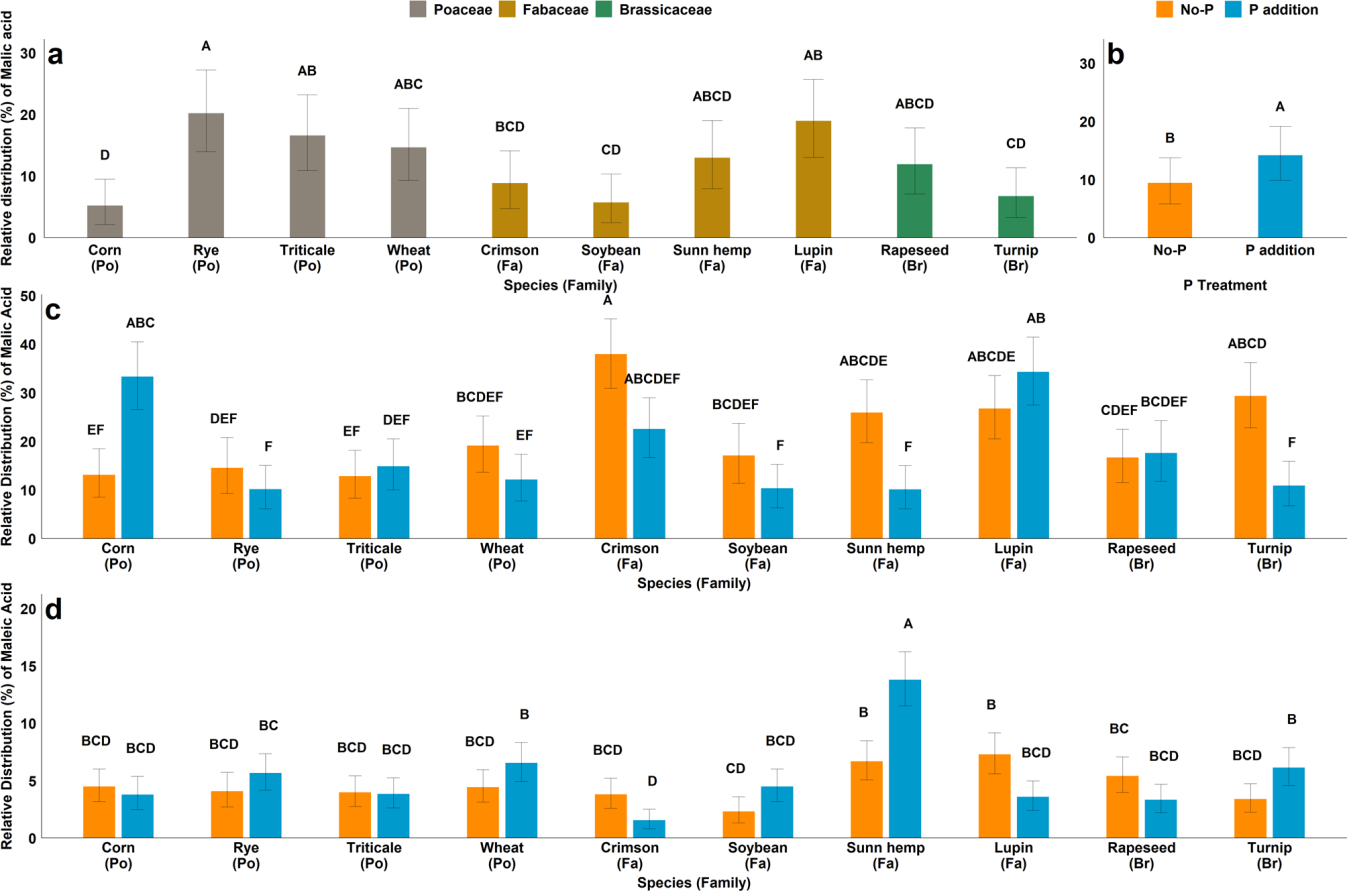
(**a**) main effect of species on the distribution of Malic acid at 35 days (**b**) main effect of P on the distribution of Malic acid at 35 days (**c**) effect of species × P interaction on the distribution of Malic acid at 70 days (**d**) effect of species × P interaction on the distribution of Maleic acid at 70 days. (Po = *Poaceae*, Fa = *Fabaceae*, Br = *Brassicaceae*) Bar with different letters indicate significant differences between treatments.

At 35 days, oxalic, malic, and fumaric acids comprised, on average, 75% of the measured total organic acids (Table S1). The relative contribution of malic acid was affected by both species (*p* = 0.016) and P treatment (*p* = 0.040) (Table S2), however, the treatments did not affect relative contributions for any other LMWOA at 35 days. Malic acid made up a larger portion of the total LMWOA release for rye, lupin, triticale, and wheat (15–20%) and made up a lesser portion of LMWOA release for turnip, soybean and corn (5–7%) (Fig. 2a). Phosphorus addition increased the relative contribution of malic acid to total LMWOA (Fig. 2b). At 70 days, oxalic, malic, and fumaric acids comprised, on average, 88% of the measured total organic acids (Table S1). The relative contribution of malic acid at 70 days was affected by the species × P interaction (*p* = 0.033) (Table S2). The addition of P increased the relative contribution of malic acids for corn whereas it decreased it for sunn hemp (from 26 to 10%) and turnip (Fig. 2c). The relative contribution of maleic acid was also affected by the species × P interaction (*p* = 0.045) (Table S2) where P addition increased the relative contribution of maleic acid in the total LMWOA release from sunn hemp but had no effect for other species (Table S2) (Fig. 2d). The relative contributions of citric and tartaric acids to total LMWOA release at 70 days were affected by species (p = < 0.001), where these acids made up 1.1–1.5% of total LMWOA for sunn hemp and lupin compared to only 0.4–0.7% for other species (Fig S2). However, our study found greater contribution of maleic acid than commonly found citric, tartaric, and succinic acid.

### Water extractable phosphorus (WEP)

**Fig. 3:**
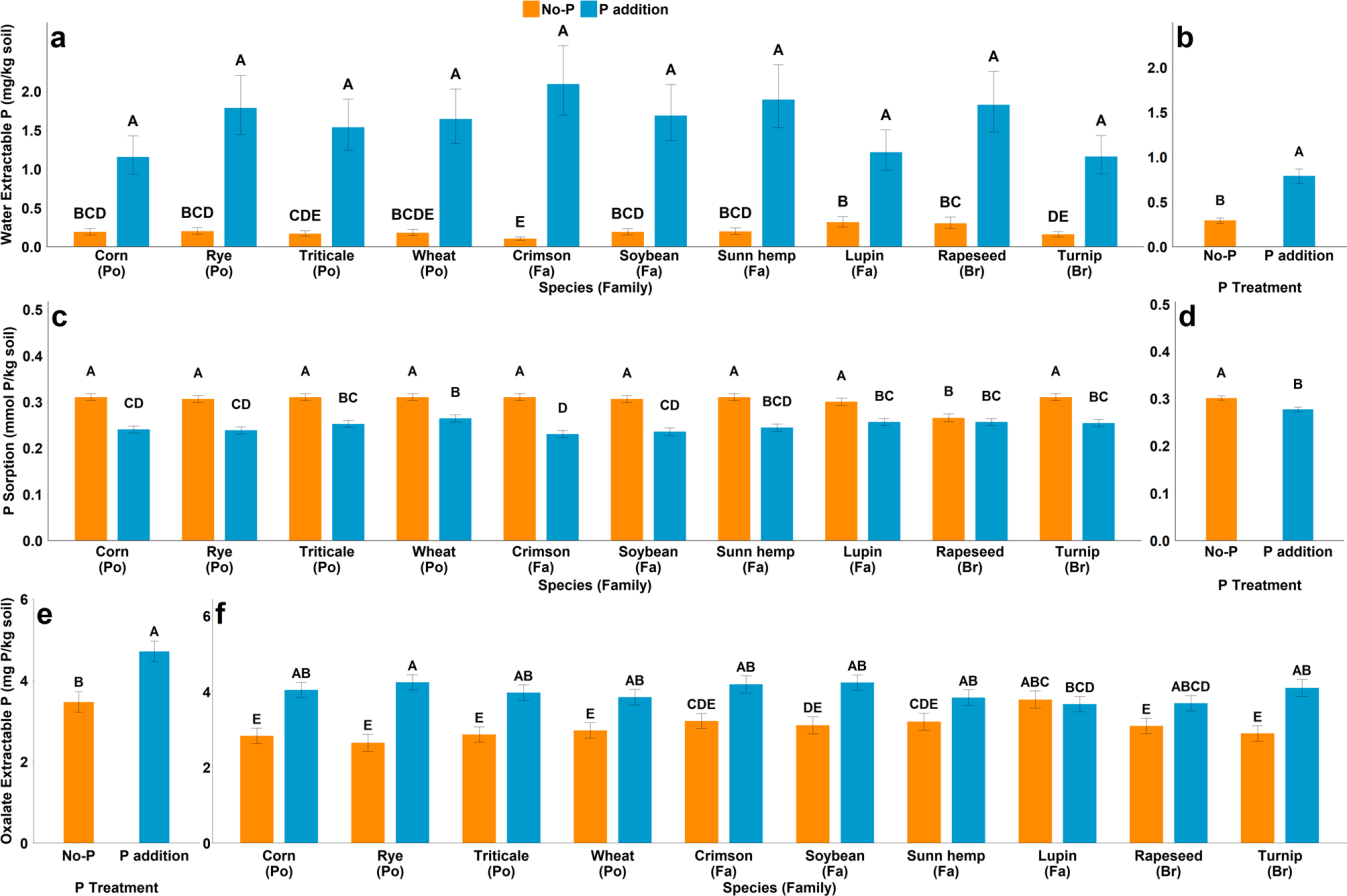
(**a**) effect of species × P interaction on water extractable P in mg P kg^−1^ soil at 35 days (**b**) main effect of P treatment on water extractable P in mg P kg^−1^ at 70 days (**c**) effect of species × P interaction on P sorption in mmol P kg^−1^ soil at 35 days (**d**) main effect of P treatment on P sorption in mmol P kg^-1^ soil at 70 days (**e**) effect of P on oxalate extract P at 35 days (**f**) effect of species × P interaction on oxalate extract P at 70 days. (Po = *Poaceae*, Fa = *Fabaceae*, Br = *Brassicaceae*) Bar with different letters indicate significant differences between treatments.

There was a species by P interaction for WEP at 35 days, where there was no effect of species on the WEP concentration when P was added (Table 5). However, when P was not applied, the WEP in soils with lupin and rapeseed was greater than in soils with crimson clover and turnip (Fig. 3a). No significant differences were found among plant families at 35 days as none of the contrasts comparing the species groupings as families were significant (Table S4). At 70 days, WEP concentration was only affected by the P treatment, where regardless of species, WEP concertation in soils that received P addition was more than double the WEP concentration in soils that did not receive P addition (Fig. 3b).

### P sorption

There was a significant species by P interaction on for P sorption for at 35 days, (Table 5; Fig. 3c) where species effect on adsorption was minimal except for rapeseed (*Brassicaceae*), which had less P adsorption relative to other species when no P fertilizer was added. With P, rapeseed had no difference with other species except crimson clover (*Fabaceae*) at 35 days. When P was added, crimson clover had less P sorption than wheat, triticale, lupin, rapeseed, and turnip. No significant differences in P sorption were found among plant families at 35 days. At 70 days, P sorption was affected by P treatment where there was greater P sorption in soils without P addition (Fig. 3d). Similar to 35 days, here no significant differences were found between plant families (Table 5). Based on the contrast statement, we observed a difference in the effect P on sorption on soils associated with *Poaceae* and *Fabaceae*, compared to those associated with *Brassicaceae* (Table S4).

### Oxalate extract P, Fe and Al

Oxalate extractable P was affected by the P treatment at both 35 and 70 days (Table 5) whereas no main effects of species were found. However, there was a significant species by P interaction for 70 days, where P addition increased oxalate-extractable P for all species except lupin (Fig. 3e and f). Furthermore, when no P was added, soils with lupin had greater oxalate extractable P relative to soils from nearly all other species (except crimson clover and sunn hemp). However, when P was added, soils with lupin had the least oxalate extractable P. The average oxalate extractable Fe and Al were 16 mg kg^− 1^ and 25 mg kg^− 1^ respectively, which was not affected by either species or P treatment (Table 5).

**Table 5:** ANOVA results assessing the effects of crop species and P treatment and their interactions on water extractable P (WEP), P sorption, oxalate extractable P, al and Fe in soils and soil pH at 35 and 70 days after planting.

	Water extractable *P*		*P* sorption		Oxalate Extract						Soil pH	
					*P*		Fe		Al			
Days	**35**	**70**	**35**	**70**	**35**	**70**	**35**	**70**	**35**	**70**	**35**	**70**
Species	0.330	0.17	0.078	0.343	0.747	0.735	0.129	0.703	0.129	0.709	0.232	**< 0.001**
P	**< 0.001**	**< 0.001**	**< 0.001**	**< 0.001**	**< 0.001**	**< 0.001**	0.563	0.832	0.615	0.909	0.548	**0.001**
Species × P	**0.046**	0.502	**0.002**	0.743	0.272	**0.016**	0.858	0.647	0.850	0.815	0.129	**< 0.001**

Significant effects and interactions (*p* < 0.05) are indicated in bold.

### Soil pH

After 35 days, no effect of either species or P was found on soil pH. The species × P interaction effect (*p* = < 0.001) (Table 5) was found after 70 days where pH decreased in soil without P addition where lupin decreased most followed by rapeseed and turnip (Table S5).

### Correlation among LMWOA in root exudates and soil properties

At 35 days without P addition, water-extractable phosphorus (WEP) showed a positive correlation with citric acid, maleic acid, and tartaric acid, but negatively correlated with oxalic acid (Table 6). This indicates that WEP increases with the increase in the release of citric, maleic and tartaric acid whereas it decreases with an increase in oxalic acid release in root exudates (Figure S3). However, no correlation was observed between WEP and total LMWOA. With P addition, no correlation of either total LMWOA or individual was found with soil properties. At 70 days without P addition, there was no correlation between total LMWOA or any individual organic acids either with WEP or P sorption. However, total LMWOA was positively correlated with WEP when P was added. Among the individual acids, only succinic acid was positively correlated with P sorption (Table 6).

**Table 6:** Pearson correlation (*p*) coefficient of total LMWOA and individual organic acids with water extractable P, and P sorption where significant results are marked with asterisk (*).

Correlation	Water extractable *P*				*P* sorption			
	35 days		70 days		35 days		70 days	
	No-P	P addition	No-P	P addition	No-P	P addition	No-P	P addition
Total LMWOA	− 0.24	− 0.23	0.05	0.77**	0.04	0.17	− 0.19	− 0.39
Citric acid	0.63*	− 0.15	0.44	− 0.48	0.09	0.28	− 0.09	0.48
Malic acid	0.38	− 0.09	0.23	− 0.34	0.16	0.53	0.13	0.49
Oxalic acid	− 0.68*	0.08	− 0.35	0.56	0.24	− 0.5	− 0.35	− 0.56
Tartaric acid	0.63*	− 0.15	0.29	− 0.46	0.09	0.28	0.45	0.47
Succinic acid	0.51	0.03	− 0.02	− 0.31	0.27	0.24	0.57	0.73*
Maleic acid	0.90***	− 0.08	0.54	− 0.08	− 0.21	− 0.33	0.37	0.22
Fumaric acid	0.27	0.09	0.01	0.01	0.31	0.02	0.53	0.51
Formic acid	0.13	0.12	0.00	− 0.31	0.31	− 0.21	− 0.21	− 0.21

Not significant (ns) *p* = > 0.05, * *p* < 0.05, **p = < 0.01; and ***p = < 0.001.

## Discussion

### Concentration and distribution of LMWOAs in root exudates

We found that plant species released different amounts and compositions of organic acids, with clear differences between species in different families. Prior studies have found that citric acid is the dominant LMWOA released by roots^50–52^, which contrasts our findings that oxalic acid was the main LMWOA in root exudates and only a small fraction was citric acid. However, most of those studies were conducted either with sterilized sand or in hydroponics with only lupin or other legume species, which usually released greater amounts of citric acid. Similar to findings in our study, Montiel-Rozas et al.^53^ observed oxalic > malic > fumaric > citric acid, with those organic acids comprising most of the total LMWOA. They also found that *Fabaceae* released greater amounts of LMWOA than *Poaceae*. They observed higher concentrations of citric acid in experiments conducted with sand, whereas oxalic acid was predominant in experiments using soils. The study of Mimmo et al.^54^ also found greater concentration of oxalic acid with soil than with soil-sand mixtures. This helps explain why our study detected greater concentrations of oxalic acid compared to studies conducted in sand or hydroponic systems. The difference is likely due to soil interactions, as citric acid has a strong affinity for soil solid phases, such as Fe and Al oxides and hydroxides^55^. It is possible that citric acid was adsorbed onto these solid phases, reducing its recovery and detectability in the root exudate samples. However, species-specific LMWOA composition may vary when plants are grown under natural environmental conditions influenced by different climatic factors.

We found that adding P to soil decreased LMWOA concentration in root exudates in some species, but not all; with distinct differences between plant families. Prior studies have found that plant roots increase LMWOA release in P-deficient soils as a P acquisition strategy^56,57^. However, contradictory results have been observed, where LMWOA concentrations increased with P addition due to the priming effect of P addition on soil microbial activity in soils with high organic matter content. Our study was conducted on soils with comparatively low organic matter content, thus explaining why we did not observe the priming effect as seen by Chen et al.^58^ and Touhami et al.^59^. The decrease in LMWOA release in response to P addition was greater for corn, rye, triticale, and crimson clover compared to other species. This indicates that in the low P soil, these species were likely releasing organic anions to increase their P acquisition^27,59,60^. Although the LMWOA release from crimson clover responded to P application, soybean and lupin, which also belong to the *Fabaceae* family, did not respond to P treatments in terms of LMWOA release. This may be because certain *Fabaceae* species release LMWOAs in response to other soil factors, such as micronutrient deficiencies^61^, rather than P availability. Species in *Brassicaceae* family were not responsive to P addition because the LMWOA concentration tended to remain high even with P addition. This indicates that *Brassicaceae* species may be releasing LMWOA in response to other environmental conditions.

### Soil phosphorus availability

We initially expected that an increase in LMWOA release would enhance P mobilization through ligand-promoted dissolution of mineral-bound P, thereby increasing the concentration of WEP in the soil solution and potentially improving its availability for plant uptake. However, at 35 days, no correlation was observed between total LMWOA and WEP. There were, however, specific organic acids, including citric acid, maleic acid, and tartaric acid, that were positively correlated with WEP in the absence of P addition (Table 6). Among these acids, citric acid (more specifically, the citrate anion) can interact with soil Fe and Al oxides via ligand-promoted dissolution due to its high affinity for Fe³⁺ and Al³⁺, with stability constants of 10^11.5^ and 10^7.5^, respectively^62,63^. This may reduce P sorption and enhance WEP. This effect was particularly evident in lupin, which released higher concentrations of citric acid (Table S6) and showed elevated WEP under P-deficient conditions. Tricarboxylic citric acid may play a primary role in influencing WEP, while dicarboxylic tartaric and maleic acids were both correlated with citric acid (*R* = 0.64 and *R* = 1.00 respectively) indicating potential for shared mechanisms of production and release within the plant. However, their lower desorption potential compared to citric acid^64^ limits their direct impact on WEP. This aligns with the effects (p = < 0.001) of species and P treatments on citric acid release observed in our study (Table S2).

We observed a significant (*p* < 0.001) effect of species and P on oxalic acid concentrations (Table S2), which were negatively correlated with WEP (Figure S3). While oxalic acid can enhance P availability through ligand-promoted dissolution and competitive adsorption, it may also form stable complexes with Fe and Al in the soil solution. These complexes can adsorb onto Fe and Al oxides, forming inner-sphere complexes and modifying surface charges. This may facilitate the formation of ternary Fe-oxalate-P complexes as these complexes act as sorbing sites for soluble P, thereby reducing P availability in the soil solution over time. Cao et al.,^61^ reported that oxalate-modified iron-based adsorbents increased P adsorption by 90% and Liang et al.^65^ also found a six-fold increase in ferrihydrite’s P adsorption capacity in the presence of oxalic acid. Although these studies used engineered materials, the mechanisms likely extend to naturally occurring iron (hydr)oxides in soils, suggesting that oxalate can enhance P adsorption by altering iron oxide surface properties or forming ternary complexes. These results underscore the importance of specific LMWOAs-particularly citric and oxalic acids-in regulating P availability during early plant growth stages, rather than total LMWOA concentration alone^1,66^.

At 70 days, with P addition, we observed an increase in WEP that correlated with total LMWOAs (Table 6). It is possible that under P addition, P from nutrient cycling remains in solution, while the previously released organic acids occupy sorption sites, preventing additional P adsorption. Our findings show that WEP dynamics are influenced by time factors, P treatment, and the composition of LMWOAs in root exudates^67^. Therefore, under certain circumstances, the release of LMWOAs in root exudates can enhance P availability in soils by increasing WEP.

Phosphorus adsorption in soils is strongly influenced by the availability of sorption sites. In P-deficient soils, these sites offer greater opportunities for P adsorption, as reflected in our study, where P adsorption was higher in soils without P addition compared to P-amended soils^68,69^. In our study, the total detected LMWOAs were primarily composed of dicarboxylic acids, which are less effective at competing with P for sorption sites compared to tricarboxylic acids like citric acid. This is due to the lower number of carboxylic groups in dicarboxylic acids, which reduces their ability to promote competitive desorption. Consequently, the limited efficacy of dicarboxylic acids in competitive sorption likely explains the lack of correlation between total LMWOA concentrations and P adsorption. Our methodology, which focuses on collecting root exudates rather than directly extracting organic acids from the soil, may have contributed to the limited detection of tricarboxylic acid like citric acid. Although minor concentrations of citric acid were detected in the root exudates, and its levels were influenced by plant species and P addition, it is possible that a portion of the citric acid reacted rapidly with soil solid phases, such as Fe and Al oxides, immediately upon release. This aligns with citric acid’s established role in reducing P sorption. This rapid reaction would reduce its availability in the collected root exudates, complicating its detection and potentially underestimating its role in influencing P sorption in soils. The study by Mimmo et al.^54^ and Trevisan et al.^70^ similarly concluded that tricarboxylic acids are potentially adsorbed onto soil solid phases when experiments are conducted with soils. At 35 days, we observed an interaction (*p* = 0.002) (Table 4) between plant species and P addition. In the absence of P addition, rapeseed exhibited the lowest sorption among the studied species (Fig. 3c). This may be attributed to the relatively high concentration of maleic acid released by rapeseed as compared to other species (Table S7). Maleic acid has a pKa₂ value of 6.23^71^, which may have caused maleic acid to preferentially occupied sorption sites, leaving fewer available for P sorption during the competitive sorption process. As a result, P sorption decreased in our single-point P sorption study. This effect was also evident in the WEP measurements for soils where rapeseed was grown (Fig. 3a), which were the highest among the species studied, second only to lupin. Interestingly, turnip released the highest concentration of LMWOAs but did not reduce P sorption as rapeseed did, despite both belonging to the *Brassicaceae* family. This suggests that specific mechanisms of P acquisition and utilization may differ among species within families. The release of LMWOAs does not always result in reduced P sorption, as supported by the lack of correlation between total LMWOA release and P sorption. When P was added, competition between P and organic anions for sorption sites in the soil solution was expected. This was evident in our results, where the addition of P led to lower P sorption in certain plant species, namely crimson clover, corn, rye, and soybean than other species. The P sorption followed the order: crimson clover < corn < rye < soybean. This pattern aligned with the release order of oxalic acid release, one of the most dominant organic acids, which can reduce P sorption through competitive adsorption (Table S7). Among the organic acids detected in this study, oxalic acid had the lowest pKa values (pKa₁ = 1.26, pKa₂ = 4.21) compared to other dicarboxylic acids. Due to its low pKa, oxalic acid readily dissociates in soil solutions. As a result, it competes with soluble P for sorption sites, thereby reducing P sorption when P is added into the system. Consequently, this competition led to a decrease in overall P sorption. This phenomenon was further reflected in our findings, where oxalic acid in root exudates played a crucial role in controlling P availability in soils. This suggests that during the early growth stages of plants, oxalic acid may enhance P availability when P is added or present in high concentrations, primarily through competitive adsorption mechanisms.

By 70 days, we observed no significant effect of plant species on P adsorption. However, our treatments influenced the concentrations of several individual organic acids, suggesting that factors other than total LMWOA might play a more prominent role in affecting P sorption at this growth stage.

Cover crops provide numerous agronomic and environmental benefits, yet their use has been associated with unintended increases in dissolved reactive phosphorus (DRP) loss from agricultural fields. It is possible that cover crops have a greater impact on solubilizing P in the system, making it available for both plant uptake and potential loss. To maximize the benefits of cover crops while minimizing DRP loss, careful selection of species is essential. Our findings suggest that certain cover crop species capable of releasing LMWOAs in response to P-deficient conditions may improve P availability in soils with limited P content. These species could enhance P availability in P-deficient soils, where it is most needed to meet the crop demand, without increasing the risk of P loss from the soils with sufficient P concentrations. Conversely, cover crop species that release LMWOAs regardless of P treatment may inadvertently increase DRP loss in P-sufficient soils, posing a risk to water quality.

## Conclusion

We observed that the types and composition of total LMWOAs in root exudates varied among plant species. Certain crop species from the *Poaceae* and *Fabaceae* families released LMWOAs as a P acquisition strategy under P-deficient conditions, whereas species from the *Brassicaceae* family consistently released higher concentrations of LMWOAs irrespective of P treatment. In some cases, P availability was correlated with the concentration of specific organic acids during early plant growth stages under P-deficient conditions, rather than the total LMWOA concentration. However, this pattern was not consistent across all conditions, highlighting the complexity of factors influencing P availability in soils. From our study, species such as cereal rye (*Poaceae*), triticale (*Poaceae*), and crimson clover (*Fabaceae*) demonstrate promise as cover crops that would enhance P availability in low P soils without unnecessarily increasing the risk for P loss in high P soils because these species were found to release limited amounts of LMWOAs in soils with sufficient or adequate P levels. By selectively promoting the use of cover crop species that align with specific soil P conditions, agricultural systems can simultaneously improve nutrient cycling and reduce environmental risks associated with P loss.

## Supplementary Information

Below is the link to the electronic supplementary material.

Supplementary Material 1.

## Data Availability

The data that support the findings of this study are available from the corresponding author upon request.
