# Correction: Soil phosphorus availability as affected by root exudates of cover crop species

**DOI:** 10.1038/s41598-026-42433-y

**Published:** 2026-03-23

**Authors:** Tamjid Us Sakib, Nathan O. Nelson, Ganga M. Hettiarachchi, Colby J. Moorberg, Jesse B. Nippert, Susan Whitaker

**Affiliations:** 1 Department of Agronomy, Kansas State University, 2004 Throckmorton Plant Sciences Center 1712 Claflin Rd., Manhattan, KS 66506 USA; 2 Division of Biology, Kansas State University, 209 Bushnell Hall 1717 Claflin Rd, Manhattan, KS 66506 USA; 3 Department of Biochemistry and Molecular Biophysics, Kansas State University, 141 Chalmers Hall 1711 Claflin Rd, Manhattan, KS 66506 USA

Correction to: *Scientific Reports* 10.1038/s41598-025-19102-7, published online 29 September 2025

The original version of the Article contained errors in Figures 1, 2 and 3.

Due to the error during figure assembly, the incorrect values were used for wheat, rye, triticale and soybean species. Additionally, the letters above the bar for lupin with P addition in Figure 1a were changed from EGHI to FGHI. The error bars in Figure 1c were corrected from +/- 1.15 for both bars to +2.05/-1.78 and +1.07/-0.93 for no-P and P addition respectively. The data in the tables was correct at the time of publication.

The original Figure 1 and its accompanying legend appears below.

**Fig. 1:**
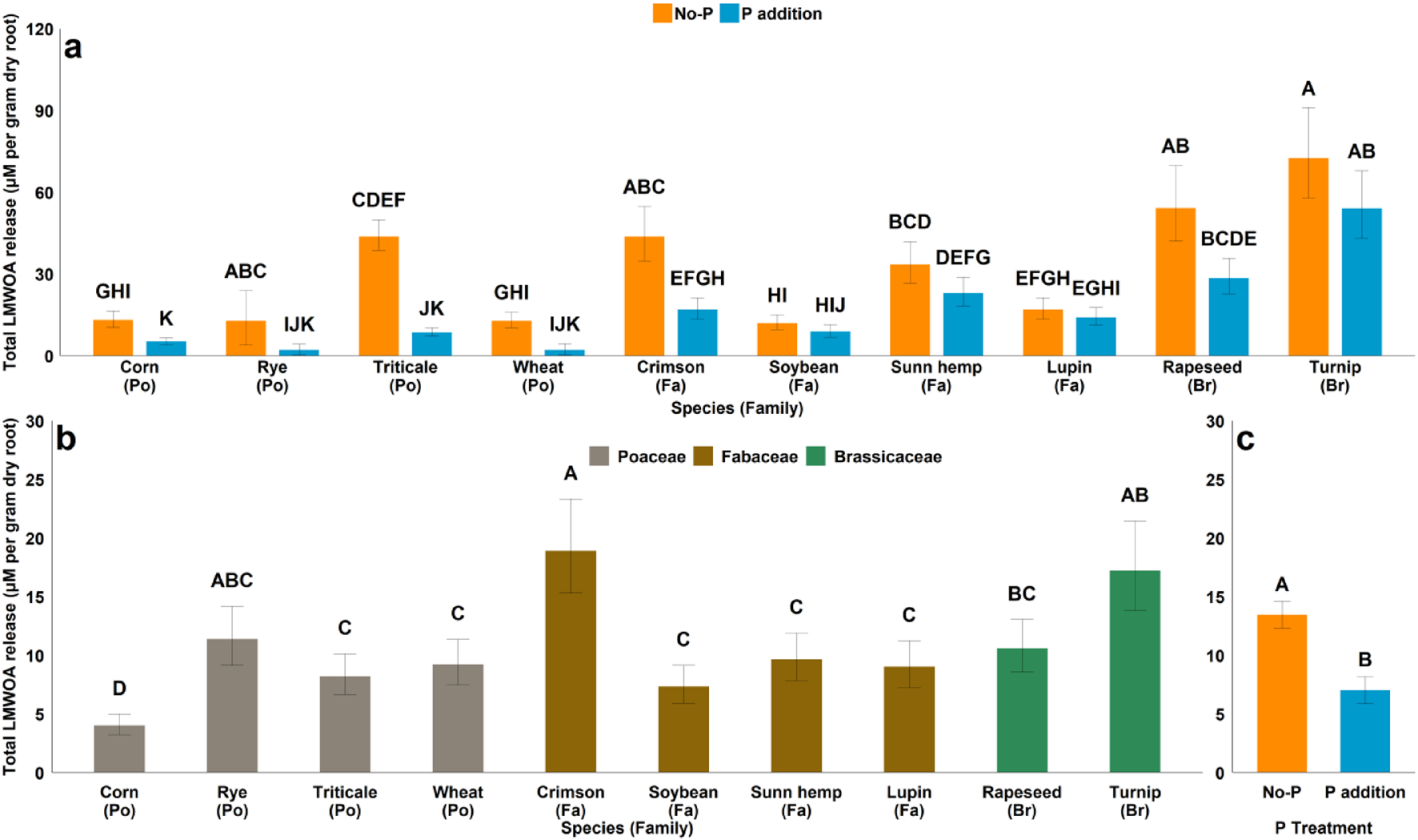
(**a**) effect of species × P interaction on total LMWOA release at 35 days after planting (**b**) main effect of species on total LMWOA release at 70 days after planting (**c**) main effect of P treatment on the total LMWOA release at 70 days after planting. (Po = *Poaceae*, Fa = *Fabaceae*, Br = *Brassicaceae*) Bar with different letters indicate significant differences between treatments.

In Figure 2a the bar values and letters were interchanged for soybean and sun hemp. In Figure 2c, bar values and letters were incorrect for soybean and sunn hemp when the figure was produced for publication. Additionally, in Figures 2c and 2d, some letters were inadvertently altered during figure preparation. The data in the tables was correct at the time of publication.

The original Figure 2 and its accompanying legend appears below.

**Fig. 2:**
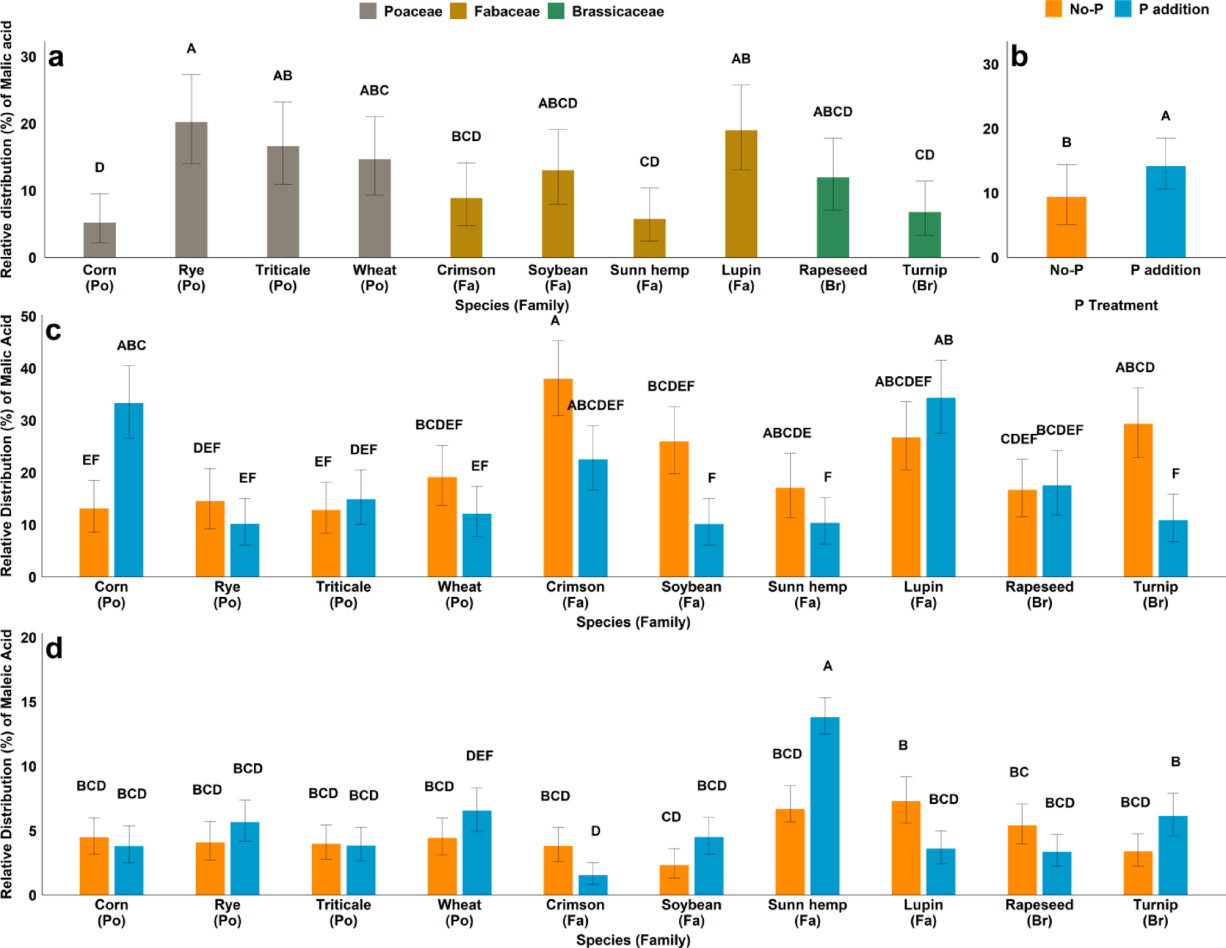
(**a**) main effect of species on the distribution of Malic acid at 35 days (**b**) main effect of P on the distribution of Malic acid at 35 days (**c**) effect of species × P interaction on the distribution of Malic acid at 70 days (**d**) effect of species × P interaction on the distribution of Maleic acid at 70 days. (Po = *Poaceae*, Fa = *Fabaceae*, Br = *Brassicaceae*) Bar with different letters indicate significant differences between treatments.

Figure 3 contained errors in Figure 3c and Figure 3e where some letters were inadvertently altered during figure preparation. The data in the tables was correct at the time of publication.

The original Figure 3 and its accompanying legend appears below.

**Fig. 3:**
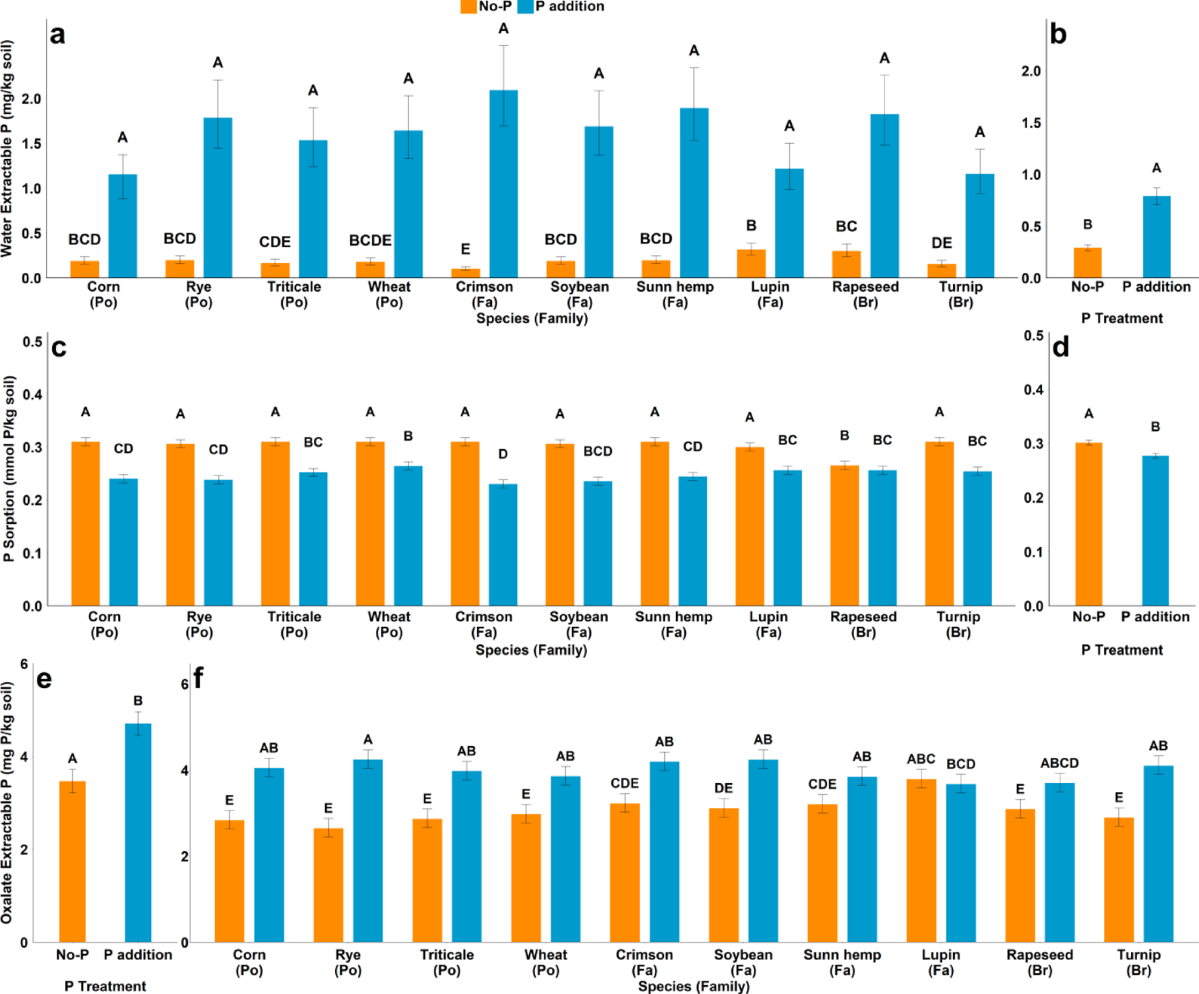
(**a**) effect of species × P interaction on water extractable P in mg P kg^−1^ soil at 35 days (**b**) main effect of P treatment on water extractable P in mg P kg^−1^ at 70 days (**c**) effect of species × P interaction on P sorption in mmol P kg^−1^ soil at 35 days (**d**) main effect of P treatment on P sorption in mmol P kg^-1^ soil at 70 days (**e**) effect of P on oxalate extract P at 35 days (**f**) effect of species × P interaction on oxalate extract P at 70 days. (Po = *Poaceae*, Fa = *Fabaceae*, Br = *Brassicaceae*) Bar with different letters indicate significant differences between treatments.

The original Article has been corrected.

