# Supplementary material for: Soil phosphorus availability as affected by root exudates of cover crop species
Source: Sci Rep. 2025 Sep 29;15:33443. doi: 10.1038/s41598-025-19102-7 (PMC12480450; doi:10.1038/s41598-025-19102-7)
Supplement: Supplementary file 1 — Supplementary Material 1. [file 41598_2025_19102_MOESM1_ESM.docx]

**Supplementary material**

**Soil phosphorus availability as affected by root exudates of cover crop species**

Tamjid Us Sakib^1^, Nathan O. Nelson^1^, Ganga M. Hettiarachchi^1^, Colby J. Moorberg^1^, Jesse B. Nippret^2^ and Susan Whitaker^3^

1. Kansas State University

Department of Agronomy

2004 Throckmorton Plant Sciences Center

1712 Claflin Rd. Manhattan, KS, 66506

1. Kansas State University

Division of Biology

209 Bushnell Hall

1615 Claflin Rd., Manhattan, KS 66506

1. Kansas State University

Department of Biochemistry and Molecular Biophysics

317 Ackert Hall

1717 Claflin Rd., Manhattan, KS 66506

**Table S1** Percentage distribution (%) of individual LMWOA in total LMWOA

| Individual LMWOA | 35 days | 70 days |
| --- | --- | --- |
| Citric | 2.02 | 0.84 |
| Malic | 14.70 | 22.88 |
| Oxalic | 42.32 | 39.22 |
| Tartaric | 1.23 | 0.54 |
| Succinic | 0.02 | 0.02 |
| Maleic | 8.44 | 5.55 |
| Fumaric | 18.49 | 35.15 |
| Formic | 16.39 | 23.13 |
| Combination of oxalic, malic and fumaric | 75 | 90.08 |

**Table S2** Probabilities (p values) from ANOVA F-tests for fixed effects of species, P fertilizer addition (P), and their interaction on the distribution of individual organic acids at 35 and 70 days after planting.

| **LMWOA** | **Citric** | | **Malic** | | **Oxalic** | | **Tartaric** | | **Succinic** | | **Maleic** | | **Fumaric** | | **Formic** | |
| --- | --- | --- | --- | --- | --- | --- | --- | --- | --- | --- | --- | --- | --- | --- | --- | --- |
| **Days** | **35** | **70** | **35** | **70** | **35** | **70** | **35** | **70** | **35** | **70** | **35** | **70** | **35** | **70** | **35** | **70** |
| Species | 0.070 | **<.001** | **0.016** | **0.013** | 0.07 | 0.884 | 0.130 | **<.001** | 0.490 | 0.183 | 0.977 | **0.001** | 0.436 | 0.977 | 0.075 | 0.512 |
| P | 0.488 | 0.128 | **0.040** | 0.115 | 0.722 | 0.828 | 0.404 | 0.211 | 0.546 | 0.317 | 0.605 | 0.493 | 0.667 | 0.298 | 0.439 | 0.66 |
| Species×P | 0.566 | 0.513 | 0.408 | **0.033** | 0.830 | 0.412 | 0.724 | 0.844 | 0.382 | 0.551 | 0.434 | **0.045** | 0.504 | 0.281 | 0.263 | 0.525 |

**Note. Significant effects and interactions (p < .05) are indicated in bold.**

**Table S3** Probabilities (p values) from ANOVA F-tests for fixed effects of species, P fertilizer addition (P), and their interaction on the concentration of individual organic acids in μM/DRW at 35 and 70 days after planting.

| **LMWOA** | **Citric** | | **Malic** | | **Oxalic** | | **Tartaric** | | **Succinic** | | **Maleic** | | **Fumaric** | | **Formic** | |
| --- | --- | --- | --- | --- | --- | --- | --- | --- | --- | --- | --- | --- | --- | --- | --- | --- |
| **Days** | **35** | **70** | **35** | **70** | **35** | **70** | **35** | **70** | **35** | **70** | **35** | **70** | **35** | **70** | **35** | **70** |
| Species | **<0.001** | **<0.001** | **0.001** | **<.001** | **<.001** | **<.001** | **<.001** | **0.020** | **<.001** | 0.187 | **<.001** | 0.122 | **<.001** | 0.985 | **<.001** | 0.583 |
| P | **0.007** | **<.001** | 0.766 | **0.001** | **<.001** | **<.001** | **<.001** | 0.717 | **<.001** | 0.314 | 0.094 | 0.403 | **<0.000** | 0.140 | **<.001** | 0.772 |
| Species×P | **0.017** | 0.671 | 0.120 | 0.**008** | 0.466 | 0.326 | 0.**007** | 0.869 | 0.**002** | 0.551 | 0.112 | 0.098 | **0**.**013** | 0.464 | **0.002** | 0.754 |

**Note. Significant effects and interactions (p < .05) are indicated in bold.**

**Table S4:** Probabilities (p values) for Contrast comparing different plant types and effect of P on the water extractable P (WEP) and P sorption, at 35 and 70 days after planting.

| Water extractable P | | | P sorption | |
| --- | --- | --- | --- | --- |
| Days | **35** | **70** | **35** | **70** |
| Po vs Fa | 0.545 | 0.077 | 0.209 | 0.06 |
| Po vs Br | 0.619 | 0.000 | 0.12 | 0.23 |
| Fa vs Br | 0.99 | 0.047 | 0.586 | 0.723 |
| Effect of P; Po vs Br | 0.439 | 0.953 | **0.004** | 0.758 |
| Effect of P; Po vs Fa | 0.651 | 0.516 | 0.539 | 0.459 |
| Effect of P; Fa vs Br | 0.257 | 0.558 | **0.001** | 0.764 |

**^Abbreviations: P = Phosphorus, Po =^ *^Poaceae,^* ^Fa =^ *^Fabaceae^* ^Ba =^ *^Brassicaceae^***

**^Note. Significant effects and interactions (p < .05) are indicated in bold^**

**Table S5** Soil pH after 35 days and 70 days

| Species | 35 days | 70 days |
| --- | --- | --- |
| Corn | 7.15abc | 6.95bcd |
| Rye | 7.16abc | 7.13ab |
| Triticale | 7.21abc | 7.13ab |
| Wheat | 7.25a | 7.19a |
| Crimson clover | 7.16abc | 7.0bcd |
| Sunn hemp | 7.19abc | 6.93cd |
| Soybean | 7.14bc | 6.92cd |
| Lupin | 7.12c | 6.84d |
| Rapeseed | 7.17abc | 6.88d |
| Turnip | 7.22ab | 6.92cd |
| Control without species | - | 7.23a |

**^Note: Different letters indicate significant differences between soils pH where the species were grown^**

**Table S6** Concentration and Percentage contribution (%) of citric acid in total LMWOA at 35 days

| Species | Concentration (μM) | | Percentage contribution (%) | |
| --- | --- | --- | --- | --- |
|  | No-P | P addition | No-P | P addition |
| Corn | 1.13 | 0.50 | 0.56 | 0.60 |
| Rye | 0.57 | 0.05 | 1.12 | 0.52 |
| Triticale | 0.13 | 0.08 | 1.09 | 1.08 |
| Wheat | 0.11 | 0.10 | 1.46 | 1.00 |
| Crimson clover | 0.23 | 0.16 | 1.93 | 1.17 |
| Sunn hemp | 0.22 | 0.15 | 1.82 | 1.49 |
| Soybean | 0.05 | 0.05 | 0.52 | 1.05 |
| Lupin | 1.70 | 2.36 | 6.18 | 5.18 |
| Rapeseed | 0.04 | 0.13 | 0.52 | 1.22 |
| Turnip | 0.06 | 0.10 | 0.85 | 0.88 |

**Table S7** LMWOAs released from each different plant species at 35 and 70 days in µM per dry gram root weight

| Species | Citric | Malic | Oxalic | Tartaric | Succinic | Maleic | Fumaric | Formic |
| --- | --- | --- | --- | --- | --- | --- | --- | --- |
| No-P at 35 days | | | | | | | | |
| Corn | 1.8862 | 2.8622 | 5.8617 | 0.8662 | 0.0030 | 0.7430 | 7.7502 | 2.6214 |
| Crimson | 0.9001 | 6.3989 | 20.9217 | 0.5495 | 0.0090 | 2.1997 | 14.9434 | 8.5471 |
| Lupin | 2.3748 | 4.9809 | 4.0538 | 1.0671 | 0.0043 | 4.4077 | 7.2004 | 2.9028 |
| Rapeseed | 0.1884 | 3.7911 | 19.2135 | 0.2490 | 0.0051 | 6.9331 | 11.2847 | 4.7579 |
| Rye | 2.2252 | 19.9553 | 13.7610 | 1.0898 | 0.0084 | 4.3205 | 5.9955 | 2.9704 |
| Soybean | 0.0731 | 1.1552 | 5.1237 | 0.0719 | 0.0015 | 0.9222 | 3.8934 | 2.7897 |
| Sun hemp | 0.7882 | 7.5634 | 16.3366 | 0.4786 | 0.0080 | 2.5246 | 12.6262 | 15.9865 |
| Triticale | 0.3250 | 2.7421 | 9.1661 | 0.2150 | 0.0041 | 1.5075 | 12.3384 | 4.1884 |
| Turnip | 0.6063 | 4.2093 | 38.7509 | 0.5691 | 0.0124 | 3.4644 | 51.0168 | 14.7908 |
| Wheat | 0.1534 | 0.8203 | 5.2611 | 0.1108 | 0.0022 | 0.8668 | 5.7011 | 2.3307 |
| P addition at 35 days | | | | | | | | |
| Corn | 0.0543 | 0.5098 | 3.8487 | 0.0552 | 0.0012 | 0.6520 | 1.7627 | 0.6779 |
| Crimson | 0.1555 | 1.5716 | 4.0582 | 0.0990 | 0.0017 | 0.9452 | 3.4772 | 1.4521 |
| Lupin | 2.3647 | 6.8105 | 2.9694 | 1.0558 | 0.0036 | 1.7309 | 5.9007 | 2.9281 |
| Rapeseed | 0.1273 | 1.8166 | 3.5726 | 0.0868 | 0.0018 | 0.8773 | 2.5230 | 1.1453 |
| Rye | 0.0458 | 2.2384 | 3.8211 | 0.0515 | 0.0011 | 0.3969 | 1.0516 | 0.9845 |
| Soybean | 0.2971 | 1.2208 | 3.7196 | 0.1603 | 0.0023 | 1.1231 | 5.0583 | 5.7021 |
| Sun hemp | 0.1465 | 1.8047 | 3.0256 | 0.0951 | 0.0021 | 1.1812 | 2.9762 | 1.5494 |
| Triticale | 0.0797 | 3.0145 | 3.6431 | 0.0662 | 0.0014 | 0.6558 | 1.0675 | 0.9291 |
| Turnip | 0.1016 | 1.1064 | 3.6614 | 0.0756 | 0.0017 | 1.2516 | 3.3360 | 1.6134 |
| Wheat | 0.1046 | 3.2563 | 3.6324 | 0.0769 | 0.0016 | 1.0073 | 2.8287 | 1.5130 |
| No-P at 70 days | | | | | | | | |
| Corn | 0.0385 | 0.3878 | 2.2602 | 0.0221 | 0.0033 | 0.5582 | 0.7414 | 2.9054 |
| Crimson | 0.1666 | 1.6763 | 9.7692 | 0.0953 | 0.0140 | 2.4125 | 3.2047 | 12.5578 |
| Lupin | 0.0928 | 0.9339 | 5.4426 | 0.0531 | 0.0078 | 1.3441 | 1.7854 | 6.9961 |
| Rapeseed | 0.0939 | 0.9445 | 5.5043 | 0.0537 | 0.0079 | 1.3593 | 1.8057 | 7.0756 |
| Rye | 0.1211 | 1.2185 | 7.1015 | 0.0693 | 0.0102 | 1.7537 | 2.3296 | 9.1286 |
| Soybean | 0.0443 | 0.4455 | 2.5961 | 0.0253 | 0.0037 | 0.6411 | 0.8516 | 3.3371 |
| Sunn hemp | 0.1219 | 1.2263 | 7.1469 | 0.0697 | 0.0103 | 1.7649 | 2.3445 | 9.1870 |
| Triticale | 0.0836 | 0.8411 | 4.9021 | 0.0478 | 0.0070 | 1.2106 | 1.6081 | 6.3014 |
| Turnip | 0.1057 | 1.0636 | 6.1985 | 0.0605 | 0.0089 | 1.5307 | 2.0334 | 7.9678 |
| Wheat | 0.0678 | 0.6826 | 3.9779 | 0.0388 | 0.0057 | 0.9824 | 1.3049 | 5.1134 |
| P addition at 70 days | | | | | | | | |
| Corn | 0.0133 | 0.1339 | 0.7806 | 0.0076 | 0.0011 | 0.1928 | 0.2561 | 1.0034 |
| Crimson | 0.0931 | 0.9366 | 5.4584 | 0.0533 | 0.0078 | 1.3480 | 1.7906 | 7.0165 |
| Lupin | 0.0719 | 0.7235 | 4.2164 | 0.0411 | 0.0061 | 1.0413 | 1.3832 | 5.4200 |
| Rapeseed | 0.0644 | 0.6479 | 3.7756 | 0.0368 | 0.0054 | 0.9324 | 1.2386 | 4.8534 |
| Rye | 0.0544 | 0.5473 | 3.1897 | 0.0311 | 0.0046 | 0.7877 | 1.0464 | 4.1002 |
| Soybean | 0.0342 | 0.3445 | 2.0077 | 0.0196 | 0.0029 | 0.4958 | 0.6586 | 2.5809 |
| Sunn hemp | 0.0503 | 0.5058 | 2.9479 | 0.0288 | 0.0042 | 0.7280 | 0.9670 | 3.7894 |
| Triticale | 0.0395 | 0.3976 | 2.3173 | 0.0226 | 0.0033 | 0.5723 | 0.7602 | 2.9788 |
| Turnip | 0.0650 | 0.6540 | 3.8116 | 0.0372 | 0.0055 | 0.9413 | 1.2504 | 4.8996 |
| Wheat | 0.0425 | 0.4273 | 2.4900 | 0.0243 | 0.0036 | 0.6149 | 0.8168 | 3.2008 |


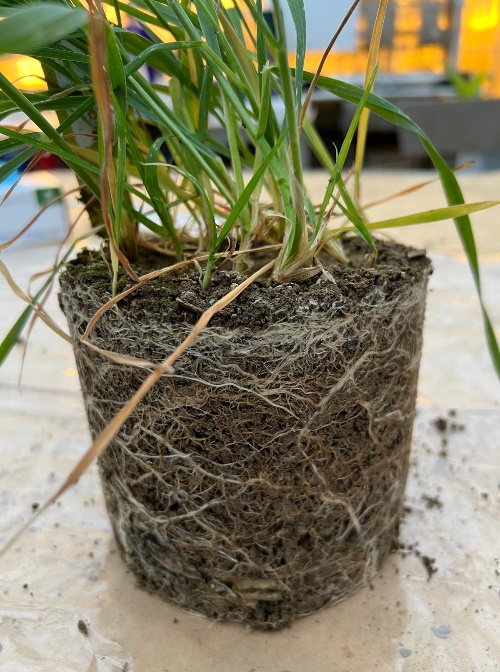


**Fig. S1. A dense root network was observed at the end of the experiment, showing root proliferation throughout the entire soil volume in the pot.**


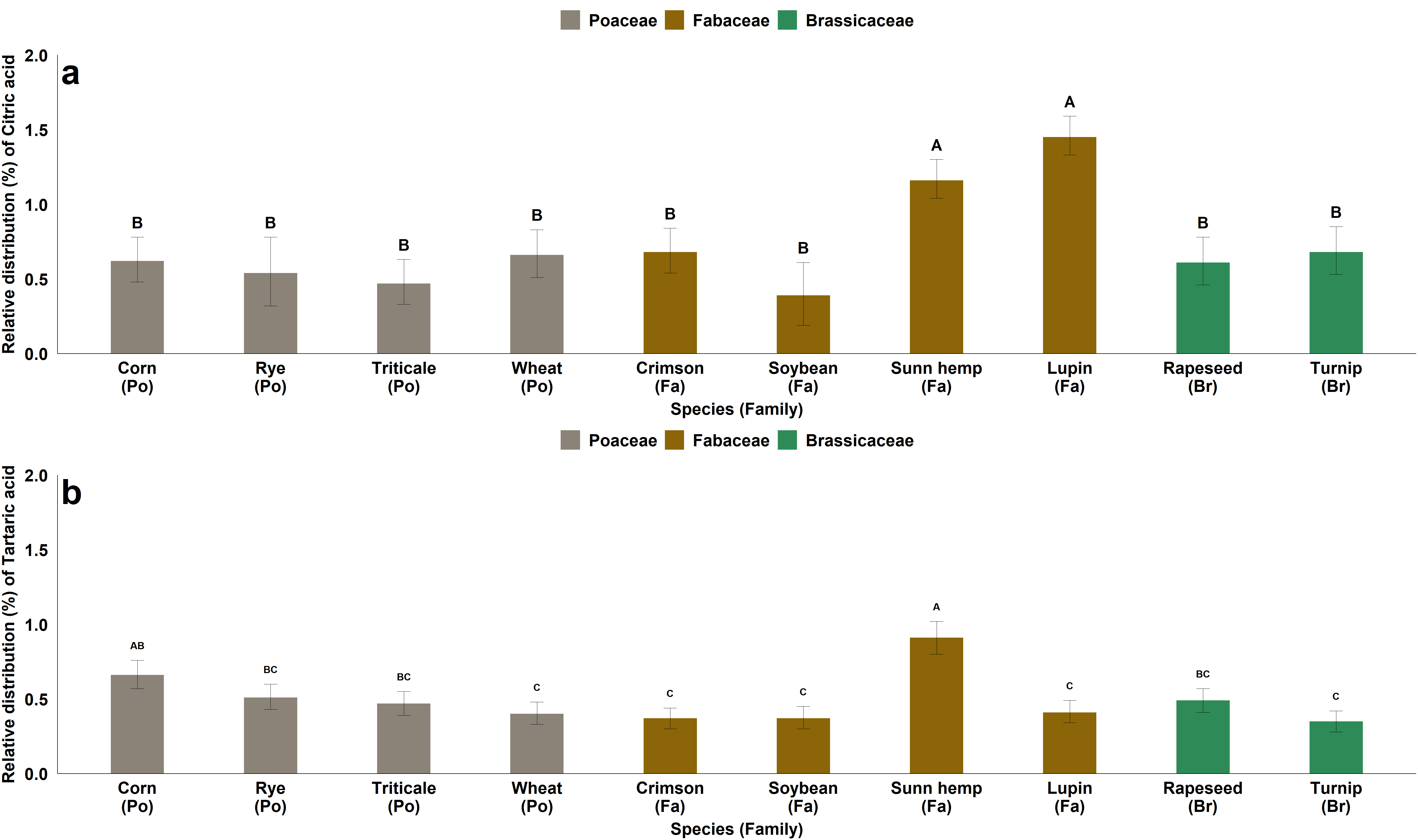


**Fig S2: (**a) Main effect of species on the distribution of Citric acid at 70 days **(**a) Main effect of species on the distribution of Tartaric acid at 70 days. (Po=*Poaceae*, Fa=*Fabaceae*, Br= *Brassicaceae*) Bar with different letters indicate significant differences between treatments.


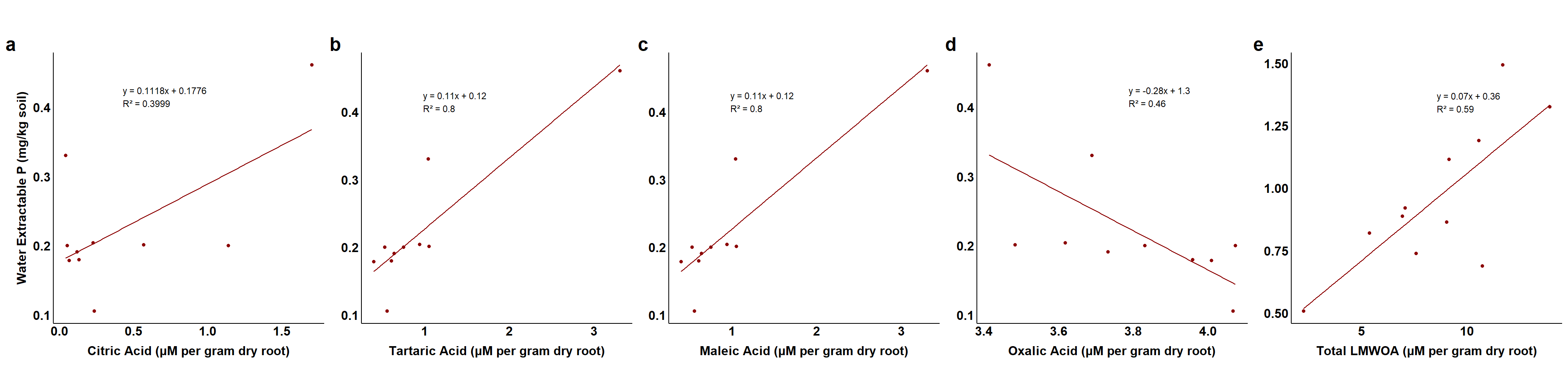


**Fig. S3** at 35 days, correlation between (a) water extractable P (WEP) and citric acid (b) water extractable P (WEP) and tartaric acid (c) water extractable P (WEP) and maleic acid (d) water extractable P (WEP) and oxalic acid (e) at 70 days, water extractable P (WEP) and Total LMWOA release at Pearson correlation coefficient at p > 0.05.
